# Cellular profile of the peritumoral inflammatory infiltrate in squamous cells carcinoma of oral mucosa: Correlation with the expression of Ki67 and histologic grading

**DOI:** 10.1186/1472-6831-8-25

**Published:** 2008-09-02

**Authors:** Fabricio LD Vieira, Beatriz J Vieira, Marco AM Guimaraes, Fernando M Aarestrup

**Affiliations:** 1 UERJ, Rio de Janeiro, RJ Brazil, Dom André Arcoverde Foundation, Valença/RJ, Brazil; 2 Dom André Arcoverde Foundation, Valença/RJ, Brazil; 3 Laboratory of Immunopathology and Experimental Pathology-CBR UFJF, Juiz de Fora, MG Brazil; 4 Dentistry School of UFJF, Juiz de Fora, MG Brazil; 5 UERJ, Rio de Janeiro, RJ Brazil; 6 Laboratory of Immunopathology and Experimental Pathology-CBR UFJF, Juiz de Fora, MG Brazil; 7 Dentistry School of UFJF, Juiz de Fora, MG Brazil, Dom André Arcoverde Foundation,Valença/RJ, Brazil

## Abstract

**Background:**

Squamous cells carcinoma is the most important malignant tumor with primary site in the oral cavity and, given the great exposure of mucosa and lips to the etiologic factors of this neoplasm, its incidence is high. Investigation of the prognostic determinants is significant for the expectations of treatment proposal and cure of the patient. The local immune response represented by peritumoral inflammatory infiltrate is a possible prognostic factor.

**Methods:**

In this study, oral mucosa samples of squamous cells carcinoma were analyzed, separated according to their histological classification as well as the phenotypical profile of the cells comprising the peritumoral inflammatory infiltrate was investigated by immunohistochemical method, in addiction, the cell proliferation index via protein Ki67 expression was determinated.

**Results:**

The T lymphocytes made up most of this inflammatory infiltrate, and among these cells, there was a predominance of T CD8 lymphocytes relative to the T CD4 lymphocytes. The B lymhocytes were the second most visualized leucocyte cell type followed by macrophages and neutrophils. The immunohistochemical assessment of Ki-67 positive cells revealed a greater expression of this protein in samples of undifferentiated squamous cells carcinoma.

**Conclusion:**

The results suggest that the cellular immune response is the main defense mechanism in squamous cells carcinoma of oral mucosa, expressed by the large number of T lymphocytes and macrophages, and that the greatest intensity of local response may be associated with the best prognosis.

## Background

Malignant neoplasms of the mouth comprise a heterogeneous group of lesions that differ among themselves as to risk factors, clinical aspects, and histologic origin. Among the different tumors of this group, squamous cells carcinoma is the most prevalent histologic type and represents most cases of diagnosed oral cancer [1-4]. Despite prevention campaigns of several governmental health care programs and expansion of the network of specialized professionals who prioritize the diagnosis of premalignant lesions of the mouth in the different health care services currently active, 7% of the world's population is affected by oral cancer, and it is, therefore, a worldwide health concern [4].

Squamous cells carcinoma originates in the epithelium that lines the mouth. This fact is extremely important when we consider the constant exposure of the oral cavity to local intrinsic stimuli associated with the increased risk of this type of cancer. Among the main risk factors, smoking, chronic alcoholic beverage consumption, and chronic mechanical traumas – such as maladapted dental prostheses and fractured teeth – stand out. Furthermore, including primary cancer of the lips, especially of the inferior portion, we should consider continuous exposure to solar radiation as a significant extrinsic factor, especially in leukodermic individuals. Besides local intrinsic factors and extrinsic factors, the patient's genetic predisposition and immune response are determining factors in the risk for cancer [2,4-10].

In addition to the intensification in prevention activities, several studies have been developed to establish the determining factors of the prognosis for these lesions with the objective of minimizing morbidity and mortality caused by the existing malignant lesion. Many authors have investigated a possible correlation between the patient's immune response, neoplastic behavior, and prognosis [5,11,12].

Meneses et al., (1998) demonstrated, in oral squamous cells carcinoma samples, a possible association between tumor size, area of invasion, angiogenesis, and the phenotypical characterization of the peritumoral inflammatory infiltrate predominantly comprised by T lymphocytes and B lymphocytes. Coussens et al., (1999) also found in carcinomas of oral mucosa an association between the predominance of mast cells in the peritumoral infiltrate and a greater development of the stromal angiogenesis, which would provide adequate blood supply for neoplastic nutrition, and consequently, a poorer prognosis.

Along with the evaluation of the peritumoral inflammatory infiltrate and its products, the study of tumoral cellular kinetics, its regulating mechanisms and its inter-relationship with growth factors, oncogenes and anti-oncogenes has also been the target of several studies [8,15-19].

Among the cellular events that directly determine the tumor's clinical progress, cellular proliferation is significant, i.e., disorders in the number of cells resulting from dysfunctions in the mitotic cycle [20,21].

The proliferative activity of any tissue or neoplasm can be determined by its growth rate using antibodies directed against specific antigens expressed by proliferating neoplastic cells, allowing the simultaneous analysis of cell proliferation and histology [13,22,23]. The Ki67 molecule has been the antigenic marker of choice, since it does not suffer much influence from internal and external factors, and its nuclear expression during a defined period of the cell's cycle represents an advantage in its use as a biological marker of mitotic activity [24-27].

Recently it was demonstrated that Ki-67 gene suffers "over expression" in epithelial cells of pre-malignant and malignant oral lesions.

In this study the cell proliferation index was evaluated by means of the expression of protein Ki67 and the phenotypical profile of cells that comprise the peritumoral inflammatory infiltrate in samples of squamous cells carcinoma of oral mucosa. The results demonstrated a vital participation of the population of T lymphocytes in the composition of the inflammatory infiltrate associated with the neoplasic area. These results also suggest a correlation between the intensity of the peritumoral inflammatory reaction and the proliferation of tumor cells.

## Methods

Samples of oral mucosa squamous cells carcinoma from treatment naïve patients submitted to diagnostic incisional biopsies. Paraffin-blocked samples (n = 30) were obtained from the archives of the Pathological Anatomy Laboratory of the Medical School of the Dom André Arcoverde Foundation – Valença/RJ.

The blocks were processed histologically to obtain slices 4 μm thick and stained with routine hematoxylin and eosin (HE).

Slides were evaluated by two different examiners, and the tumors were classified histologically according to the International Classification of Diseases for Oncology (ICD-O/2000). Samples were divided into the following groups:

Group 1: well-differentiated squamous cells carcinoma of oral mucosa (n = 10)

Group 2: moderately differentiated squamous cells carcinoma of oral mucosa (n = 10)

Group 3: undifferentiated squamous cells carcinoma of oral mucosa (n = 10).

### Histomorphometry of the inflammatory infiltrate

In order to quantify the peritumoral inflammatory infiltrate, Scion Image software and FotoScan software were used for image capture and morphometry of the inflammatory infiltrate. All samples were captured in full extent and fields (original magnification 100×) with inflamed areas around the tumor were measured semi-automatically, with manual selection of the inflamed region and computed analysis of the selected area.

Based on the analysis of each sample, simple arithmetic means the inflamed area per microscopic field were obtained and the results were expressed in percentage of inflamed peritumoral area per group.

### Immunohistochemical Analysis

Slices with 4 μm thickness arranged on silanized slides (3-aminopropyltriethoxysilane; Sigma Chemical, Co; USA), were deparaffinized in a 60°C chamber, and sequentially hydrated in passages through xylol, absolute alcohol, 70% alcohol, and distilled water.

The classic avidin-biotin peroxidase anti-peroxidase complex method was used in studying the samples. Samples were submitted to antigen retrieval with immersion of the fragments in 0.001 M citrate buffer, PH 6.0, and peroxidase block with 3% hydrogen peroxide and further incubated for 1 hour with the primary antibodies specified on Table 1. Next, they were incubated with secondary biotinylated antibodies for 30 minutes and submitted to a reaction with the avidin-biotin complex for another 30 minutes. Staining was performed by the addition of the diaminobenzidine chromogen (DAB) substrate for about 1 minute.

**Table 1:** Antibodies for immunohistochemistry analysis Antibody Marking Brand

Anti CD20	B Lymphocytes	Dako
Anti CD3	T Lymphocytes	Dako
Anti CD8	Regulatory T Lymphocytes	Dako
Anti CD4	Helper T Lymphocytes	Dako
Anti CD15	Neutrophils	Dako
Anti CD68	Macrophages	Dako
Anti Ki67	Cellular proliferation	Dako

Negative control of the immunohistochemical reaction was performed by omitting incubation with the primary antibody for some slices. Slices were analyzed and photomicrographs were made with the Nikon Microphot system (Tokyo, Japan).

Reactive cell count for each antibody was made by scanning the entire sample, with 400× magnification, with a count of positive cells in all fields. Results were obtained from the simple arithmetic mean and expressed in percentage of positive cells per microscopic field.

### Statistical Analysis

Statistical analysis was performed using Mann-Whitney with a p < 0.05 significance level in order to comparatively evaluate means of the parameters analyzed in the different groups of the experiment.

## Results

**Figure 1:**
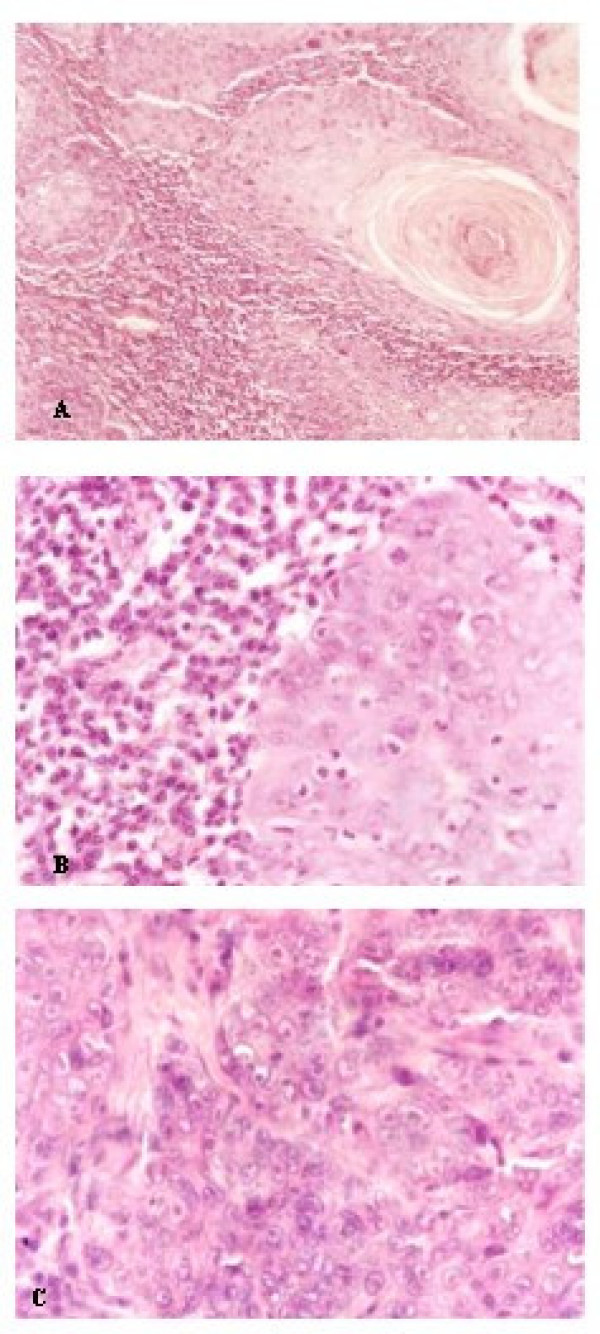
**Morphological grading of squamous cells from the buccal mucosa.** A: Well-differentiated squamous cell carcinoma of buccal mucosa. N = 10. HE stain. Original magnification 200×. B: Moderately differentiated squamous cell carcinoma of buccal mucosa. N = 10. HE stain. Original magnification 400×. C: Undifferentiated squamous cell carcinoma of buccal mucosa. N = 10. HE stain. Original magnification 400×.

**Figure 2:**
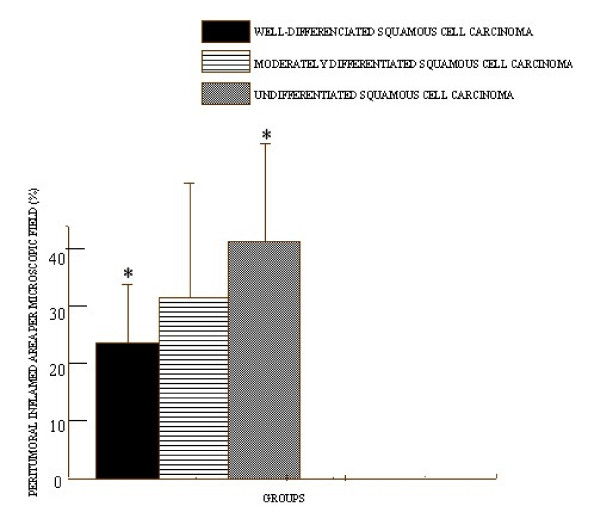
**Intensity of peritumoral inflammatory infiltrate.** Values are expressed in simple arithmetic means ± sd. Statistical significant difference was observed when compared groups 1 and 3. p ≤ 0.05.

The histopathology analysis of the slices stained with hematoxylin and eosin (HE) and submitted to morphometric analysis revealed that the mean percentage of peritumoral inflamed area per microscopic field was 24.7% in Group 1 (samples of well-differentiated squamous cells carcinoma of the oral mucosa) (Figure 1a), 33.2% in Group 2 (samples of moderately differentiated squamous cells carcinoma of the oral mucosa) (Figure 1b), and 42.6% in Group 3 (samples of undifferentiated squamous cells carcinoma of the oral mucosa) (Figure 1c). After statistical evaluation, the amount of peritumoral inflamed area showed a statistically significant difference when Groups 1 and 3 were compared (p < 0.05) (Figure 2).

In all the samples evaluated in this study, the quantitative predominance of the inflammatory infiltrate was directly associated with areas of greatest invasion into tissues subjacent to the tumor parenchyma.

The phenotypical evaluation of the peritumoral infiltrate was carried out by identification of inflammatory cell positivity, characterized by the visualization of light brown intracellular coloring, against a background counter-stained with hematoxylin in each one of the reactions for the antibodies used.

**Figure 3:**
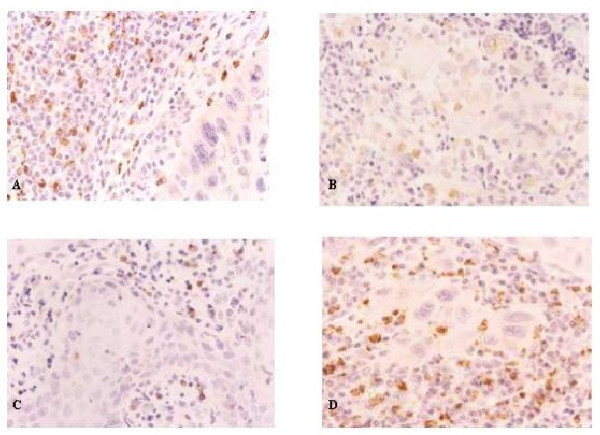
**Immunohistochemistry for phenotypical characterization of peritumoral inflammatory infiltrate.** A: lymphocytes T (CD3+ cells). Original magnification 400×. B. A: lymphocytes B (CD20+ cells). Original magnification 400× C. A: Neutrophils (CD15+cells). Original magnification 400× D. A: Macrophages (CD68+cells). Original magnification 400×.

In all groups, analysis of the immunohistochemical analysis showed the presence of an inflammatory reaction constituted predominantly by mononuclear inflammatory cells. The T lymphocytes made up most of this inflammatory infiltrate, and among these cells, there was a predominance of T CD8 lymphocytes relative to the T CD4 lymphocytes (Figure 3a). The B lymphocytes were the second most visualized leukocyte cell type (Figure 3b), followed by macrophages (Figure 3d) and neutrophils (Figure 3c).

**Figure 4:**
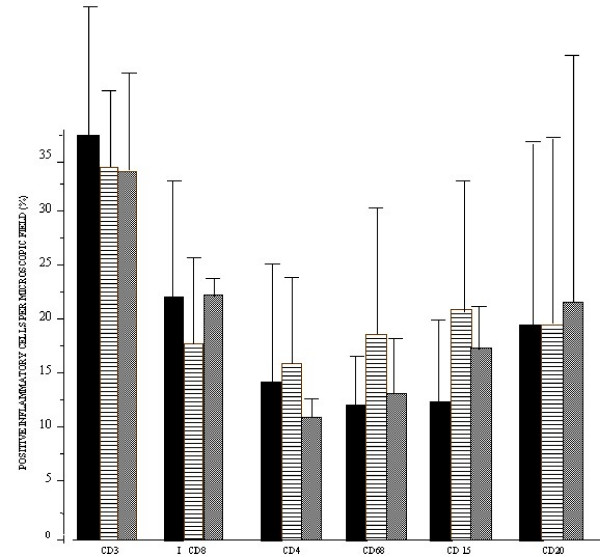
**Cellular profile of the peritumoral inflammatory infiltrate.** Values are expressed as simple arithmetic means ± sd. No statistical significant difference was observed when compared the groups. p ≤ 0.05.

There was no significant difference when leukocyte types of the three groups were compared (p > 0.05) (Figure 4).

**Figure 5:**
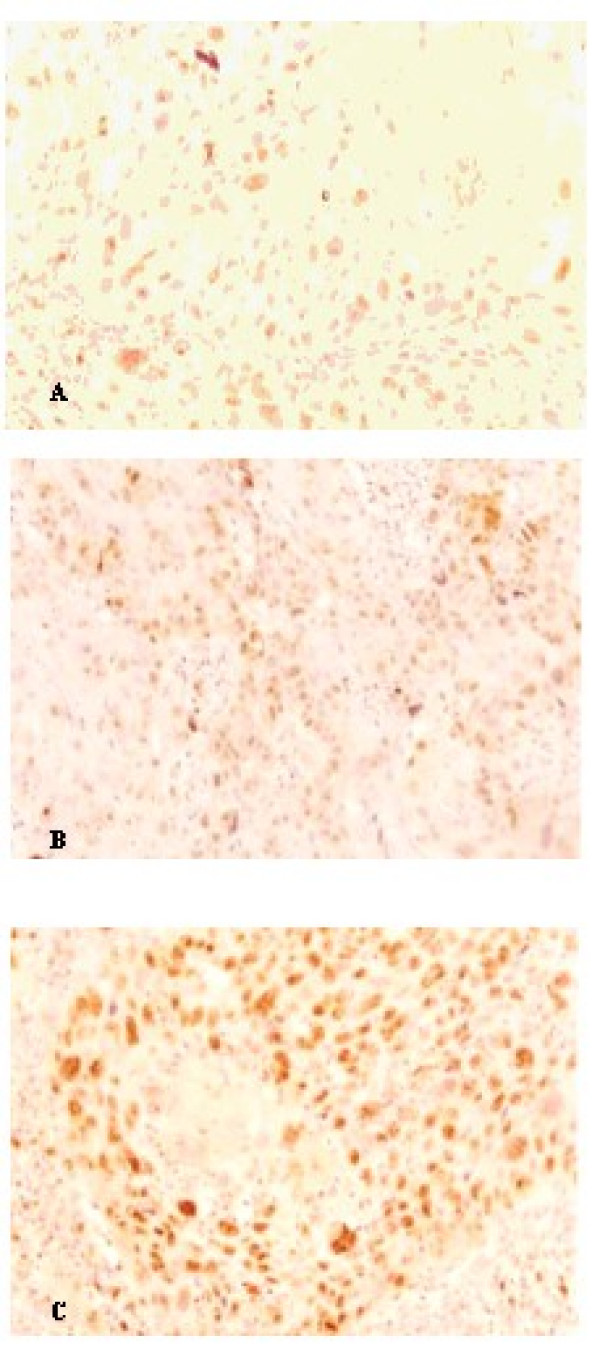
**Immunohistochemistry demonstrating expression of protein Ki67 in malignant tumor cells from samples of oral mucosa squamous cells carcinoma.** A: Well-differentiated carcinoma. Original magnification 400×. B: Moderately differentiated carcinoma. Original magnification 400×. C: Undifferentiated carcinoma. Original magnification 400×.

**Figure 6:**
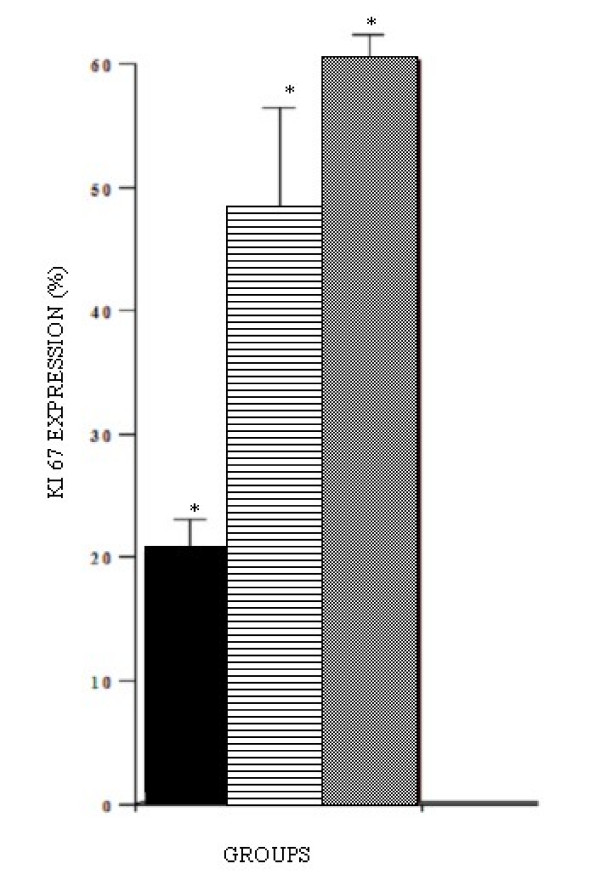
**Expression of protein Ki67 per group.** Values are expressed in simple arithmetic means ± sd. Statistical significant difference was observed when compared the groups. p ≤ 0.05.

In samples of patients from Group 1, the immunohistochemical assessment of protein Ki-67 revealed scarce immunoreactive tumor cells (20% ± 3,2) (Figure 5), while in samples of patients from Group 2, cells with positivity were more abundant (47% ± 6,3). In samples of Group 3 patients, cells marked positively represented 60.3% ± 2,8 of the malignant tumor cells. The comparison between the percentages of tumor cells positive for Ki-67 expression per microscopic field in the oral mucosa samples of the three groups showed statistically significant differences (p < 0.05) (Figure 6). Tumors from Group 3 showed a greater expression of protein Ki-67 compared to the tumors from Groups 1 and 2.

## Discussion

Squamous cells carcinoma is the most prevalent type of cancer of the oral mucosa, and represents 91% of the diagnosed cases of malignant tumors of the mouth [2,6-8].

Besides the multifactor etiology involving extrinsic and intrinsic factors, the patient's immune status is also considered to influence the risk of cancer and determine several aspects of its progression [4,9,10]. In the 1980s, Scully C. (1983) had already recognized the involvement of the immune response in the development of malignant mouth tumors, and had emphasized that future cancer treatment would be based on immunotherapy through immunomodulation.

Among the events that directly determine tumor clinical progression, those of primary importance include disorders in the number of cells resulting from mitotic cycle dysfunctions and possible failures" in host's immune evasion of the tumors – assessed by the intensity of the peritumoral inflammatory infiltrate, inflammatory cell composition, cytokine production by infiltrate cells, and presence of angiogenesis [11,12,14,20,21].

According to Costa L. et al., (2005) in a retrospective clinical study with 38 samples of oral squamous cells carcinoma, the TNM classification (tumor staging determined by the size of the primary cancer and the presence of metastases) correlates with the chief histopathologic characteristics of tumoral classification – degree of keratinization, nuclear pleomorphism, and intensity of peritumoral lymphocytic infiltration, and the link among these data is important in determining prognosis and choosing treatment.

The present results showed an inverse correlation between the degree of tumor differentiation and the rate of cell proliferation obtained by the expression of protein Ki67. Results similar to those found in this study are also suggested by Costa et al., (2005) in oral carcinoma, Glen et al., (2006) in malignant pancreatic lesions, Deans et al., (2006) in gastro-esophageal cancers, and Cai et al., (2006) in transitional cell carcinoma of the bladder. The undifferentiated tumors showed an accentuated expression of protein KI-67. Aguiar (1996) demonstrated that the mitotic index increases progressively from normal peritumoral oral mucosa towards tumor areas, as well as in areas of greatest tissue invasion [7]. Pich et al., (2004) in a retrospective study with malignant lesions of the mouth cavity, salivary glands, pharynx, and larynx, observed that the proliferative activity investigated by different methods – such as the AgNORs Index determination and MIB-1 and Ki67 expression by immunohistochemistry – is clinically relevant and valid for proposing treatment and defining prognosis. In this paper, the histologic characteristics exhibited by the malignant tumor were associated with the clinical aggressiveness by the analysis of the malignant grade related to possible determinants of lesion prognosis – local immunologic profile and malignant cell proliferation.

According to the results obtained from morphometry of the peritumoral infiltrate, the average percentage of inflamed area per microscopic field was greater in the undifferentiated tumors (Group 3) when compared to the averages of the moderately differentiated tumor samples (Group 2), followed by the well-differentiated tumor samples (Group 1). In evaluating these data, we noted that there is a correlation between the highest degree of malignity and the greatest inflammatory intensity.

The characterization of the peritumoral infiltrate composition did not reveal a difference between the leukocyte types of the three groups, determined from their grades, but interestingly, the quantities of each cell type were equally proportional in these groups. We note that in all samples, the total number of T lymphocytes and macrophages predominated over the quantification of plasmocytes, characterizing the patient's local reaction as a predominantly cell type immune response.

In addition to the evaluation of the peritumoral inflammatory infiltrate intensity and its cellular components, some of the major determinants of tumor aggressiveness and possible predictors of prognosis are alterations in cell proliferation, i.e., abnormalities in the number of cells resulting from mitotic cycle dysfunctions. This neoplastic proliferative activity can be determined by the growth rate by means of Ki67 expression [18-21,28-31].

The comparison among the percentages of tumor cells positive for the expression of Ki-67 per microscopic field in the oral mucosa samples of the three groups showed statistically significant differences. The samples from Group 3 (undifferentiated squamous cells carcinoma of the oral mucosa) displayed a greater expression of cells marked positively, followed by the samples of patients from Group 2 (moderately differentiated squamous cells carcinoma of the oral mucosa), and from Group 1 (well-differentiated squamous cells carcinoma of oral mucosa).

As to leukocyte characterization, Sica et al., (2006) reported evidence associated to the predominance of the macrophage population in the peritumoral infiltrate and a greater promotion of tumoral angiogenesis, attributing to these cells a pro-tumoral role, and consequently, a poor prognosis.

In the samples studied of oral carcinoma, the evaluation of peritumoral inflammatory cells demonstrated the importance of cellular immunity in the local antineoplastic response due to the presence of a population predominantly composed of T lymphocytes and macrophages, albeit with no relevant differences as to the infiltrate composition according to the histological grading.

Recently, in samples of invasive breast cancer, we investigated the possible correlation between the intensity of the peritumoral inflammatory infiltrate and the degree of tumor differentiation [32]. We point out that when we analyzed patients with malignant tumors of the same grade, those who progressed satisfactorily showed a more intense peritumoral inflammatory response, while those who experienced tumor relapses and metastatic dissemination developed a less intense peritumoral inflammatory response.

## Conclusion

In this study, the parallel between intensity of the inflammation and the patient's prognosis has not yet been fully clarified, and further research is needed. The distribution of the oral mucosa carcinoma samples into groups was made according to histologic grading. The results suggest a positive correlation between inflammatory response intensity and the degree of tumor differentiation. Therefore, undifferentiated tumors present a greater development of the peritumoral inflammatory process when compared to moderately differentiated and well-differentiated tumors. Nevertheless, in order to define this variable of inflammatory response intensity as a prognostic factor in oral cancer, other studies should be carried out so that the intensity of the local response and malignant cell proliferation in tumors of the same histopathologic classification associated to the patients' clinical progress can be evaluated.

Finally, the results presented in this study suggest that the cellular immune response is the main defense mechanism in oral mucosa squamous cells carcinoma, expressed by the large number of T lymphocytes and macrophages.

We further underscore that Ki-67 expression is related to the mitotic index and, consequently, to cellular proliferation and malignant grading of the neoplasm.

## Competing interests

The authors declare that they have no competing interests.

## Authors' contributions

FLDV and BJV participated in the designed of the study, immunohistochemical study and histopathological study. FMA and MAMG participated in the design and coordination.

## Acknowledgements

Rede mineira de bioterismo. 2824/05 – FAPEMIG; Rede mineira "TOXIFAR". 2827/05 – FAPEMIG; CNPQ

## Pre-publication history

The pre-publication history for this paper can be accessed here:


